# Hair-Pulling Does Not Necessarily Serve an Emotion Regulation Function in Adults With Trichotillomania

**DOI:** 10.3389/fpsyg.2021.675468

**Published:** 2021-07-05

**Authors:** Christine Lochner, Salome Demetriou, Martin Kidd, Bronwynè Coetzee, Dan J. Stein

**Affiliations:** ^1^South African Medical Research Council Unit on Risk and Resilience in Mental Disorders, Department of Psychiatry, Stellenbosch University, Stellenbosch, South Africa; ^2^Department of Psychology, Stellenbosch University, Stellenbosch, South Africa; ^3^Centre for Statistical Consultation, Stellenbosch University, Stellenbosch, South Africa; ^4^South African Medical Research Council Unit on Risk and Resilience in Mental Disorders, Department of Psychiatry and Neuroscience Institute, University of Cape Town, Cape Town, South Africa

**Keywords:** trichotillomania, hair-pulling disorder, emotion regulation, emotion dysregulation, childhood trauma, stress

## Abstract

**Background:** Trichotillomania (TTM) has been associated with childhood trauma and perceived stress. While it has been hypothesized that hair-pulling regulate negative emotions, the relationship between childhood trauma, perceived stress, emotion regulation, and hair-pulling has not been well-studied.

**Methods:** Fifty-six adults with TTM and 31 healthy controls completed the Childhood Trauma Questionnaire (CTQ), Perceived Stress Scale (PSS), and Difficulties in Emotion Regulation Scale (DERS). Hair-pulling severity was measured with the Massachusetts General Hospital-Hair Pulling Scale. CTQ, PSS, and DERS total scores were compared across groups using ANCOVA and the correlation between hair-pulling severity and emotion dysregulation was determined. Regression analyses were used to estimate the association of CTQ and PSS totals with DERS, and to determine whether associations between predictors and dependent variable (DERS) differed across groups.

**Results:** TTM patients reported higher rates of childhood trauma (*p* <= 0.01), perceived stress (*p* = 0.03), and emotion dysregulation (*p* <= 0.01). There was no association between emotion dysregulation and pulling severity (*r* = −0.02, *p* = 0.89). Perceived stress was associated with emotion dysregulation in both groups (*p* < 0.01), and no association between childhood trauma and emotion dysregulation in either group. Perceived stress was the only significant predictor of emotion dysregulation in both groups (*F* = 28.29, *p* < 0.01).

**Conclusion:** The association between perceived stress and emotion dysregulation is not specific to TTM, and there is no association between emotion dysregulation and hair-pulling severity, suggesting that key factors other than emotion dysregulation contribute to hair-pulling. Alternative explanatory models are needed.

## Introduction

Trichotillomania (hair-pulling disorder, or TTM) is characterized by recurrent hair-pulling or -plucking resulting in hair loss, and repeated attempts to decrease or stop the behavior (American Psychiatric Association, 2013). Tension relief, gratification, or pleasure during or after pulling are reported by some with this condition, while significant distress and/or functional impairment are required for the diagnosis.

Various theoretical models of hair-pulling have been proposed (e.g., Duke et al., 2010; Arabatzoudis et al., 2017), but there is no consensus about the most suitable (Meunier et al., 2009; Duke et al., 2010; Curley et al., 2016). The emotion regulation (ER) model has received particular attention (Diefenbach et al., 2008; Roberts et al., 2013; Arabatzoudis et al., 2017). This model hypothesizes that hair-pulling is a maladaptive coping mechanism to regulate negative emotional states, e.g., providing stimulation when feeling bored, or relieving tension when stressed or anxious (Shusterman et al., 2009; Duke et al., 2010; Grant et al., 2015; Roberts et al., 2015; Curley et al., 2016).

Consistent with the ER model, hair-pulling may occur in response to trauma and stressors (Gershuny et al., 2006). The relationship between trauma/stressors and TTM may not be a direct causal one, but rather indirect, with hair-pulling acting as a maladaptive mechanism to regulate negative affect associated with traumatic or stressful events. Evidence for this model includes findings that childhood adversity is higher in TTM patients compared to healthy controls (Lochner et al., 2002; Gershuny et al., 2006; Özten et al., 2015), and that stressors are associated with hair-pulling (Grant et al., 2015). Furthermore, individuals with TTM may have more difficulty constructively regulating their emotions than healthy controls (Shusterman et al., 2009; Arabatzoudis et al., 2017), and treatment strategies focused on ER, such as dialectical behavioral therapy (DBT) and acceptance and commitment therapy (ACT), may be efficacious for TTM (Woods et al., 2006b; Keuthen et al., 2012).

Notably, hair-pulling may, in turn, have negative consequences for the individual, with hair-pulling often leading to negative emotions such as guilt, depression and/or anxiety (e.g., Diefenbach et al., 2008; Shusterman et al., 2009; Grant et al., 2015; Houghton et al., 2016). Indeed, TTM is highly comorbid with affective disorders such as depression and anxiety disorders, which are also characterized by impaired regulation of negative affect (American Psychiatric Association, 2013).

Several gaps in the literature remain. In particular, few studies have explored the relationship between childhood trauma, perceived stress, emotion dysregulation, and hair-pulling severity. This study aimed to assess the ER model by investigating associations between these variables in TTM. Specific objectives were to compare rates of childhood trauma, perceived stress and emotion dysregulation between TTM cases and controls, to investigate the association between emotion dysregulation and hair-pulling severity, and finally, to assess the associations between childhood trauma, perceived stress, and emotion dysregulation in TTM and in healthy controls.

## Materials and Methods

Participants with TTM were recruited from a wide range of sources (e.g., media advertisements, community based primary care practitioners, and psychiatrists and psychologists). Healthy controls were recruited from the community and our university campus through media advertisements. Participants were included if they were between the ages of 18 and 70 years. Assessments were done by a clinical psychologist over a 6-month period, during 2018.

Fifty-six adults with primary TTM (mean age: 36.04 [SD: 14.55] years; 91.1% female) and 31 sex-matched healthy controls (mean age: 29.61 [SD: 9.78] years), 93.5% female) were interviewed. The Structured Clinical Interview for DSM-IV-TR Axis-I Disorders (SCID-I) (First et al., 1998) and the Structured Clinical Interview for Obsessive Compulsive Spectrum Disorders (SCID-OCSD) (Du Toit et al., 2001) were administered by a clinical psychologist, and the diagnosis of TTM was based on DSM-5 criteria. The Massachusetts General Hospital-Hair Pulling Scale (MGHHPS) was used to assess pulling severity over the past week (Keuthen et al., 1995). All participants completed the 28-item Childhood Trauma Questionnaire (CTQ) to rate the severity of emotional abuse and neglect, physical abuse and neglect and sexual abuse (Bernstein et al., 2003), the 10-item Perceived Stress Scale (PSS) to assess the degree to which situations in one's life are appraised as stressful over the past month (Cohen et al., 1983), and the 36-item Difficulties in Emotion Regulation Scale (DERS) to assess current emotion regulation ability (Gratz and Roemer, 2004). The Cronbach alphas (α) of the CTQ subscale totals ranged between 0.33 and 0.87. Although the Cronbach alpha for the physical neglect subscale was low (0.33), this subscale total correlated well with the other CTQ subscale totals (correlations not shown here), and the Cronbach alpha of the CTQ total was high (0.77), suggesting that the internal consistency of the CTQ was satisfactory. The PSS items were highly correlated with one another, and the α of the PSS total was high (0.89). In terms of the DERS subscale and full-scale totals, α was also high, ranging between 0.74 and 0.94 (total DERS score: α = 0.84). Use of the DERS total score in analyses is supported by the significant and generally strong loadings of DERS items on the general factor, providing support for the validity of a total score as a representation of emotion regulation ability (e.g., Hallion et al., 2018).

TTM patients were significantly older than controls in our sample (*p* = 0.006). Analysis of covariance was, with age as covariate, thus used to compare the CTQ, PSS, and DERS total scores between the two groups. Pearson correlation was used to investigate the association between emotion dysregulation (DERS total) and hair-pulling severity (MGHHPS total). Multivariable linear regression analysis was used to estimate the relationship between total scores on the CTQ and PSS, respectively (independent variables), and DERS total (dependent variable) in TTM, controlling for the presence of lifetime mood and anxiety disorders. The latter included major depressive disorder, bipolar disorder, social anxiety disorder, generalized anxiety disorders, and panic disorder (with or without agoraphobia). The regression analysis to estimate the relationship between total scores on the CTQ and PSS (independent variables), respectively, and DERS total (dependent variable), was repeated in healthy controls. Finally, a linear regression (GLM procedure) was conducted to determine whether associations between the predictors (totals of the CTQ and PSS) and the dependent variable (DERS total) differed across the two groups.

The Statistica package version 13.3 for Windows (Statsoft, Tulsa, Oklahoma) was used as statistical program. The Sobel test was done using the R package “multilevel” v2.6.

The study procedures were carried out in accordance with the Declaration of Helsinki. The Institutional Review Board of Stellenbosch University approved the study. All participants provided informed consent after a full explanation of the study.

## Results

Of the 56 individuals with TTM, 27 (48.21%) presented with one or more lifetime anxiety disorders, and 29 (51.79%) with one or more lifetime mood disorders. Healthy controls were free of any current or lifetime psychiatric disorders.

Childhood trauma was significantly higher in the TTM cohort (CTQ mean total 38.96, SD 13.13, SE 1.57) compared to controls (CTQ mean total 32.03, SD 7.79, SE 2.11*)* [*F*_(1, 84)_ = 4.85, *p* < = 0.01; eta-squared = 0.07 (medium)]. TTM cases also reported significantly higher perceived stress rates (PSS total mean 18.31, SD 7.43, SE 1.03) compared to controls (PSS total mean 14.47, SD 8.01, SE 1.41) [*F*_(1, 84)_ = 5.12, *p* = 0.03; eta-squared = 0.07 (medium)]. In addition, emotion dysregulation was significantly higher in individuals with TTM (DERS mean total 79.71, SD 22.15 SE 2.1) compared to healthy controls (DERS mean total 65.55, SD 21.56, SE 4.03) [*F*_(1, 84)_ = 0.03, *p* < = 0.01; eta-squared = 0.09 (medium)].

The mean MGHHPS total score was 15.9 (SD 4.3). Regarding correlation with hair-pulling severity, no significant results were found for childhood trauma (*r* = 0.06, *p* = 0.66), perceived stress rates (*r* = 0.23, *p* = 0.09), and emotion dysregulation (*r* = −0.02, *p* = 0.89).

Perceived stress (PSS mean total) was significantly and positively correlated with emotion dysregulation (DERS mean total) in TTM (*r* = 0.5, *p* < 0.01) and in healthy controls (*r* = 0.63, *p* < 0.01). Childhood trauma (CTQ mean total), on the other hand, did not correlate with emotion dysregulation (DERS mean total) in either TTM (*r* = 0.15) or controls (*r* = 0.27; both *p* > 0.05) (Table 1).

**Table 1:** Correlations between total scores on the DERS, CTQ, PSS, and age per diagnostic group^*^.

**TTM (*n* = 56) → Controls (*n* = 31) ↓**	**DERS total** **Mean (SD): 79.44 (22.15)**	**CTQ total** **Mean (SD): 38.96 (13.13)**	**PSS total** **Mean (SD): 18.31 (7.43)**	**Age** **Mean (SD): 36.04 (14.55)**
DERS totalMean (SD): 66.03 (21.56)		0.18 (*p* = 0.18)	**0.50 (*****p*** **<** **0.01)**	−0.08 (*p =* 0.55)
CTQ totalMean (SD): 32.03 (7.79)	0.21 (*p =* 0.27)		0.30 (*p =* 0.03)	0.11 (*p =* 0.42)
PSS totalMean (SD): 14.47 (8.01)	**0.63 (*****p*** **<** **0.01)**	0.39 (*p =* 0.03)		−0.26 (*p =* 0.06)
AgeMean (SD): 28.87 (9.14)	−0.04 (*p =* 0.84)	−0.16 (*p =* 0.38)	0.09 (*p =* 0.65)	

*Pearson correlations above the diagonal are for TTM patients, and below the diagonal for healthy controls;*

* *statistically significant findings (p < 0.01) are in bold type*.

Variables included in the regression model explained 25% of the variance in the DERS mean total in individuals with TTM. Controlling for lifetime mood and anxiety disorders, perceived stress level (PSS mean total) was a statistically significant positive predictor of emotion dysregulation (DERS total) in TTM (β = 0.47, *p* < 0.01) (Table 2).

**Table 2:** Multivariable linear regression analysis for emotion dysregulation as dependent variable, in the two groups separately.

**TTM (*n* = 56)**	**Standardized coefficients**	**Unstandardized coefficients**		***t***	***p***
	**β**	**β**	**Standard error of β**		
CTQ Total	−0.09	−0.16	0.24	−0.64	0.52
PSS total	0.50	1.48	0.39	3.77	<0.01^*^
Lifetime anxiety disorders	0.22	9.80	5.64	1.74	0.09
Lifetime mood disorders	0.10	4.50	5.74	0.78	0.44
**Healthy controls (*****n*** **=** **31)**	**Standardized coefficients**	**Unstandardized coefficients**		***t***	***p***
	***β***	***β***	**Standard error of** ***β***		
CTQ Total	−0.03	−0.09	0.48	−0.18	0.86
PSS total	0.65	1.77	0.45	3.90	<0.01^*^

*CTQ total, Childhood Trauma Questionnaire total score; PSS total, Perceived Stress Scale total score;*

* *statistically significant findings (p < 0.01) are in bold type*.

Repeating the regression analysis in healthy controls, childhood trauma (CTQ mean total) and perceived stress level (PSS mean total) predicted 35% of the variance in emotion dysregulation (DERS total), with perceived stress level the only statistically significant positive predictor of emotion dysregulation (β = 0.72, *p* < 0.01) (Table 2).

In the regression model (GLM), associations between predictors (totals of the CTQ and PSS) and the dependent variable (DERS total) did not significantly differ across the two groups with both group^*^CTQ and group^*^PSS interaction terms not significant (*p* = 0.92 and *p* = 0.71, respectively). Perceived stress level was the only statistically significant predictor of emotion dysregulation in both groups (*p* < 0.01).

## Discussion

The main study findings were (1) that perceived stress is associated with emotion dysregulation in both TTM and healthy controls, and (2) that although patients with TTM reported significantly higher rates of childhood trauma, perceived stress and emotion dysregulation than healthy controls, there was no association between emotion dysregulation and hair-pulling severity in TTM.

Current perceived stress was not only significantly higher in our TTM cohort compared to controls, but it was also the only variable investigated here that predicted significant variance in emotion dysregulation in TTM. Nevertheless, this association between stress and emotion dysregulation is not specific to TTM; it was found in our healthy controls, and it has been described in other conditions. For example, the stressor of social threat may lead to increased drug use in animals (e.g., Haney et al., 1995) and drug and alcohol craving and use in humans (Sinha and Li, 2007). Clinically, it makes sense to focus on the management of stressors to promote constructive emotion regulation strategies which may, in turn, lead to more favorable outcomes (Gross, 1998), irrespective of diagnostic status.

In line with earlier work, patients with TTM reported significantly higher rates of childhood trauma, perceived stress, and emotion dysregulation than healthy controls, but emotion dysregulation was not related to pulling severity. That the presence of TTM is associated with emotion dysregulation but without a dose-response relationship between TTM severity and emotion dysregulation, may indicate that the association is not precisely linear. However, the finding that there was no association between hair-pulling severity and emotion dysregulation differs from earlier work (Shusterman et al., 2009; Rufer et al., 2014; Weidt et al., 2016), perhaps due to differences in sample size (smaller samples in the current study), the varying extent of differences in emotion dysregulation rates between cases and controls (larger differences in rates of emotion dysregulation between cases and controls in earlier work), and use of different emotion dysregulation assessment scales (e.g., DERS, Affective Regulation Scale). Our finding also suggests that other factors are involved in hair-pulling. Factors specific to TTM might include excess habit formation (Gillan et al., 2016), cognitive disinhibition (Chamberlain et al., 2006) and dissociation (e.g., Lochner et al., 2004; Gupta, 2013). Childhood trauma, perceived stressors, and emotion dysregulation may also play a less specific but contributory role (e.g., Lochner et al., 2002; Woods et al., 2006a; Özten et al., 2015; Alexander et al., 2018). Further work is needed to develop and consolidate a model of hair-pulling.

There were a few limitations to this study. First, the majority of the scales used were self-report measures, which may increase the risk of response bias. Nevertheless, these questionnaires are well-established measures with sound statistical properties. Second, the majority of the sample was female, preventing generalizability to males. This is consistent with other samples however, where there is often an ~9:1 female to male ratio (Grant and Chamberlain, 2016). Third, TTM patients were older than controls in our sample (*p* = 0.006), and therefore we included age as covariate in our analyses. Notably, age had no significant association with emotion regulation in any of the cohorts, and both cohorts comprised adults mostly in their 20 or 30's, with their recall of childhood trauma (Goodman et al., 2019), perceived stress level (Osmanovic-Thunström et al., 2015), and emotion regulation strategies (Isaacowitz et al., 2017) likely not unduly influenced by age. Fourth, as noted earlier, our sample size, particularly of healthy controls, is relatively small. Despite these limitations, the present study provides additional insight into emotion regulation difficulties in TTM by exploring the relationships between emotion dysregulation, childhood trauma, perceived stress, and hair-pulling severity.

In conclusion, this preliminary investigation is one of the first to explore the applicability of the ER model in TTM. The findings that the association between perceived stress and emotion dysregulation is not specific to TTM, and that there is no association between emotion dysregulation and hair-pulling severity in TTM, suggest that key factors other than emotion dysregulation contribute to hair-pulling. Alternative explanatory models for this disorder are needed, and further research on models of hair-pulling may need to include constructs such as excessive habit formation or cognitive disinhibition. In the interim, in the clinic emphasis should be placed on addressing a range of modifiable factors associated with hair-pulling (e.g., Stein et al., 2006), including emotion dysregulation.

## Data Availability Statement

The raw data supporting the conclusions of this article will be made available by the authors, without undue reservation.

## Ethics Statement

The study protocol and informed consent forms were reviewed and approved by Health Research Ethics Committee, Stellenbosch University. The patients/participants provided their written informed consent to participate in this study.

## Author Contributions

CL, SD, BC, and DJS contributed to conception and design of the study. CL and SD organized the database. CL and MK performed the statistical analysis. CL wrote the first draft of the manuscript. All authors contributed to manuscript revision, read, and approved the submitted version.

## Conflict of Interest

The authors declare that the research was conducted in the absence of any commercial or financial relationships that could be construed as a potential conflict of interest.
